# Comment on “Comparative Effectiveness of 2 Next-Generation Scatter Radiation Shielding Systems”

**DOI:** 10.1016/j.jscai.2025.104052

**Published:** 2025-12-11

**Authors:** David G. Rizik

**Affiliations:** Cardiovascular Service Line, Banner University Medical Center, Scottsdale, Arizona

As interventional cardiology continues to advance, so too does our responsibility to protect the physicians, staff, and patients who rely on these life-saving procedures. With the introduction of enhanced radiation protection devices (ERPDs) such as EggNest, Rampart, Protego, and Radiaction, the field stands on the threshold of significant change. Yet as we evaluate these technologies, we must demand the highest standards of scientific inquiry, transparency, and clinical relevance.

## The foundation of comparative science

At the heart of impactful comparative research lies uncompromising methodological rigor. This is not simply a matter of academic preference, but a necessity; our clinical decisions and the safety of our teams hinge on data that reflect real-world practice, not abstractions or partial representations. When we evaluate new protections against radiation, we must ensure that our comparisons are fair, comprehensive, and grounded in the context of contemporary medicine.

## Critical appraisal of the phantom model study design

The recent study by Riley et al,^1^ “Comparative Effectiveness of 2 Next-Generation Scatter Radiation Shielding Systems,” offers a timely look at ERPDs in the cardiac catheterization laboratory, utilizing an anthropomorphic phantom model to simulate clinical conditions. On one hand, the authors are to be congratulated for their commitment to advancing the field of radiation protection through dedicated clinical research, as such efforts are critical for driving progress in an area of great importance to the safety of interventional teams. In addition, the use of a phantom is well established for controlled dosimetric evaluation. That said, several critical methodological concerns must be addressed to ensure the study’s findings are meaningfully interpreted and applied.

First, the comparative design did not reflect the full clinical reality of the devices under investigation. The EggNest system was evaluated in its entirety, incorporating all manufacturer-recommended components. In contrast, the Rampart system was represented solely by its Defender shield, omitting key elements such as the Bunker, Shadow, Sentry, and the more contemporary ceiling-mounted Guardian shields. These are all potentially integral to the system’s intended real-world configuration and have been proven to significantly reduce scatter radiation in prior studies.^2^^,^^3^ This “partial evaluation” of only a subset of the Rampart system potentially skews the comparative outcome, raising questions about the validity and generalizability of the results.

Additionally, the study’s setup of the Rampart Defender may have deviated from standard clinical practice, despite the manufacturer’s specific on-site training protocols for optimal placement. Misconfigurations of this nature are not trivial; they can materially impact performance and thus the study’s conclusions. For a comparative analysis to be robust and fair, both devices must be deployed as they would be in actual clinical workflows, using the full complement of shielding as per each manufacturer’s guidance.

## Measurement standards and scientific rigor

Beyond system representation, the approach to measurement warrants scrutiny. The study reported averaged scatter radiation levels across multiple positions and vertical heights. Although this approach may offer a broad overview, it does not account for the nonuniformity of radiation fields typically encountered in the catheterization laboratory. Regulatory guidance, including Nuclear Regulatory Commission Reg. Guide 8.40,^4^ recommends the use of Effective Dose Equivalent calculations with tissue weighting factors to provide a more accurate assessment of occupational risk. The omission of such calculations, as well as reporting μSv/h values without accounting for tissue attenuation or calibration factors, risks overstating actual dose and may mislead practitioners about the true protection offered by these devices.

Furthermore, the involvement of a credentialed health physicist or radiation safety professional in study design and analysis is not a mere formality. Such expertise is vital for ensuring methodological integrity, proper calibration, and the nuanced interpretation of complex dosimetric data.

## Transparency, independence, and the role of journals

Transparency and independence are nonnegotiable pillars of credible research. The presence of conflicts of interest, such as a lead author’s advisory role with a sponsoring manufacturer, does not, in itself, negate the value of a study. However, it does necessitate greater scrutiny, adherence to rigorous protocols, and, when appropriate, independent validation. Peer-reviewed journals bear a responsibility to ensure that published research reflects clinical realities, properly discloses limitations, and invites open, balanced discourse among all stakeholders.

## Premature comparisons: Where should the bar be set?

Perhaps most importantly, it is likely premature to draw head-to-head comparisons between ERPDs such as EggNest and Rampart at this stage. These technologies, while promising, are not the current standard of care in the vast majority of US catheterization labs; clinical experience is limited, and relatively few hospitals have incorporated them into their routine practice. As we are only in the early, embryonic stages of adopting enhanced radiation protection, the most scientifically meaningful comparator remains the established standard: lead aprons worn by staff and table-mounted drop-down lead shields. Just as we would not have prematurely compared Cipher to Taxus drug-eluting stents when bare metal stents were still the standard of care (prior to US Food and Drug Administration approval and broad drug-eluting stent experience), so too must we exercise scientific restraint when assessing new ERPD technologies.^5^

Future comparative studies should focus on evaluating the efficacy, usability, and cost-effectiveness of ERPDs against this current benchmark before attempting to distinguish between novel technologies that have yet to see widespread adoption or standardization. Only through this incremental, stepwise, evidence-based approach can we provide the clarity and confidence needed to guide the field forward.

## The evolving landscape: Other ERPD technologies

The emergence of devices such as Protego^6^, ^7^, ^8^ and Radiaction^9^^,^^10^ further underscores the dynamic and rapidly shifting landscape of radiation protection. As these and other technologies continue to evolve and enter the market, it becomes even more critical that our research methods keep pace, ensuring that comparisons are fair, clinically representative, and truly informative for practitioners considering adoption.

In summary, innovation in radiation protection is both necessary and welcome. But as we move forward, we must anchor our progress to the foundational principles of scientific rigor, transparency, and clinical relevance. Comparative studies must evaluate devices as they are actually implemented in clinical practice, involve appropriate expertise, and utilize measurement protocols that reflect occupational realities. Above all, we should be cautious in making premature claims about the relative merits of novel ERPDs and instead focus on establishing their value relative to the true current standard of care.

By holding our research to these standards, we ensure that advancements in radiation protection are not only innovative, but meaningful, improving safety for those who provide and receive care in the cardiac catheterization laboratory.
